# Nongenic cancer-risk SNPs affect oncogenes, tumour-suppressor genes, and immune function

**DOI:** 10.1038/s41416-019-0614-3

**Published:** 2019-12-06

**Authors:** Maud Fagny, John Platig, Marieke Lydia Kuijjer, Xihong Lin, John Quackenbush

**Affiliations:** 1 Genetique Quantitative et Evolution–Le Moulon, Institut National de la Recherche agronomique, Université Paris-Sud, Centre National de la Recherche Scientifique, AgroParisTech, Université Paris-Saclay, Paris, France; 2 Channing Division of Network Medicine, Brigham and Women’s Hospital, Boston, MA USA; 3 Harvard Medical School, Boston, MA USA; 4 Department of Biostatistics and Computational Biology, Dana-Farber Cancer Institute, Boston, MA USA; 5 Department of Biostatistics, Harvard T.H. Chan School of Public Health, Boston, MA USA; 6 Centre for Molecular Medicine Norway, University of Oslo, Oslo, Norway; 7 Department of Cancer Biology, Dana-Farber Cancer Institute, Boston, MA USA

**Keywords:** Data integration, Regulatory networks, Cancer genomics

## Abstract

**Background:**

Genome-wide association studies (GWASes) have identified many noncoding germline single-nucleotide polymorphisms (SNPs) that are associated with an increased risk of developing cancer. However, how these SNPs affect cancer risk is still largely unknown.

**Methods:**

We used a systems biology approach to analyse the regulatory role of cancer-risk SNPs in thirteen tissues. By using data from the Genotype-Tissue Expression (GTEx) project, we performed an expression quantitative trait locus (eQTL) analysis. We represented both significant *cis-* and *trans-*eQTLs as edges in tissue-specific eQTL bipartite networks.

**Results:**

Each tissue-specific eQTL network is organised into communities that group sets of SNPs and functionally related genes. When mapping cancer-risk SNPs to these networks, we find that in each tissue, these SNPs are significantly overrepresented in communities enriched for immune response processes, as well as tissue-specific functions. Moreover, cancer-risk SNPs are more likely to be ‘cores’ of their communities, influencing the expression of many genes within the same biological processes. Finally, cancer-risk SNPs preferentially target oncogenes and tumour-suppressor genes, suggesting that they may alter the expression of these key cancer genes.

**Conclusions:**

This approach provides a new way of understanding genetic effects on cancer risk and provides a biological context for interpreting the results of GWAS cancer studies.

## Background

Cancers often result from somatic mutations in oncogenes and tumour suppressors, which frequently arise due to environmental exposures such as UV light, tobacco, smoke or carcinogenic chemicals.^1–3^ Hereditary cancers represent between 5 and 10% of all cancers and are characterised by a family history of the disease, a younger than usual age of onset and a higher likelihood of primary cancers in multiple organs. They are often associated with germline alterations in oncogenes or tumour-suppressor genes.^4^ But beyond these obvious cancer ‘drivers,’ it is widely recognised that other genetic factors play a role in cancer development and progression. Genome-wide association studies (GWASes) have identified germline single-nucleotide polymorphisms (SNPs) that are associated with altered cancer risk (‘cancer-risk SNPs’). However, many SNPs identified through GWASes fall into non-genic regions, making it difficult to interpret their biological role in disease development, progression and response to therapy.^5^

The population frequency of a germline cancer-risk SNP is generally anti-correlated with its effect, calculated as the relative risk between people who carry the mutation and those who do not.^6^ Although the functions of the small number of rare variants with strong effects are well-studied, little is known about the functions of the more common risk variants with small effects that are present at intermediate frequency in the general population. Amongst the SNPs in the GWAS catalog that pass the genome-wide significance bar for association with an elevated risk for one or more cancers, most have an odds ratio <1.3, and most fall outside of genes (are located in ‘non-genic’ regions), suggesting that they may play a role in the regulation of gene expression.^6,7^

Expression quantitative trait locus (eQTL) analysis tests for associations between the genotype at a SNP locus and expression levels of a gene, and an eQTL association can provide evidence for a SNP’s regulatory role. Several studies have shown that SNPs associated with traits and diseases in GWAS are enriched for eQTLs, thus reinforcing the hypothesis that they may play a regulatory role.^8,9^ A number of studies have also found that cancer-risk SNPs influence gene expression levels.^10–13^ However, most germline cancer-risk SNPs have not been biologically characterised, and their functional impact in the general population is not known.

This gap in our understanding of cancer-risk SNPs may be due to their inherent characteristics. In addition to their small effect on the macroscopic phenotype (developing cancer), cancer-risk SNPs also usually have small effects on the expression of individual genes.^14^ Moreover, because many genes exhibit tissue-specific expression, it is difficult to characterise the regulatory role of cancer-risk SNPs that target genes not expressed in the most frequently studied tissues, such as whole blood.^14,15^ Finally, because the transformation of a healthy cell into a cancer cell is associated with many genomic and transcriptomic changes, we cannot use the studies of tumour cells to investigate the effect of the regulatory cancer-risk SNPs on pre-tumour cells.

New data sets can help shed light on the role of cancer-risk SNPs. Large-scale studies, such as the Genotype-Tissue Expression (GTEx) project, provide genomic and transcriptomic data from hundreds of individuals and dozens of non-diseased tissues,^16^ thus allowing the effects of cancer-risk SNPs to be assessed in multiple tissues, including those in which their effects are most relevant.

In this study, we used a system biology approach to characterise the regulatory role of germline cancer-risk SNPs in 13 different tissues (Supplementary Table S1) by using data from the GTEx project v6.0 . In each tissue, we performed an eQTL analysis and represented both *cis*- and *trans*-eQTLs by using a bipartite network. We then mapped both germline cancer-risk SNPs and the oncogenes and tumour-suppressor genes to these networks and used the properties of the networks to identify the biological functions and pathways that cancer-risk SNPs affect.

We find that although cancer-risk SNPs are distributed across the network, they are enriched in a small number of communities associated with immune response and recognition of pathogens, underscoring the importance of immune processes in cancer. In particular, cancer-risk SNPs preferentially map to communities enriched for genes belonging to the major histocompatibility complex (MHC), indicating a potentially greater role for immune processes in cancer risk than might have been expected. We also find that cancer-risk SNPs are overrepresented among local community hubs ('core SNPs'), by regulating multiple genes involved in the same biological function both in *cis* and in *trans*. Finally, we find that cancer-risk SNPs are preferentially located in the promoters of oncogenes and tumour-suppressor genes, and are more likely than expected by chance to influence the expression level of these cancer-related genes. This analysis demonstrates the power of using tissue-specific bipartite eQTL networks as a framework to investigate how germline SNPs can act coordinately to deregulate the expression of biological functions and can lead to an increased risk of developing cancer.

## Methods

### GTEx data preprocessing, filtering and merging

We downloaded NHGRI GTEx v6.0 imputed genotyping data and RNA-seq data (phs000424.v6.p1, 2015-10-05 release) from dbGaP (approved protocol #9112). The RNA-Seq data were preprocessed by using Bioconductor R YARN package^17^ and normalised in a tissue-aware manner by using smooth quantile normalisation Bioconductor R qsmooth package.^18^ We identified and removed GTEx-11ILO due to potential sex misannotation. We also filtered out sex chromosomes and mitochondrial genes, retaining 29,242 genes. We excluded five sex-specific tissues (prostate, testis, uterus, vagina and ovary) and grouped skin samples from the lower leg (sun exposed) and from the suprapubic region (sun unexposed) based on overall gene expression similarity between these sites. For our analysis, we only considered tissues for which we had both RNA-seq and imputed genotyping data for at least 200 individuals. Thirteen tissues met all criteria in preprocessing and were used in subsequent analyses (Supplementary Table S1).

The RNA-seq and genotyping data were mapped by the GTEx Consortium to GENCODE version 19, which was based on human genome build GRCh37.p13 (Sept 2015). We performed principal component analysis on the RNA-Seq data in each tissue, and searched for potentially confounding metadata elements by searching for those correlated with the first ten RNA-Seq principal components. For all tissues, we accounted for the site where the donor was recruited, the RNA extraction kit effects, the quality of extracted RNA, the death place, the time interval between death and start of the tissue sampling and whether or not the donor was on a ventilator immediately prior to death by using the R limma package.^19^

### eQTL mapping and bipartite network construction

For eQTL analysis, we excluded SNPs from all analyses if they had a call rate under 0.9 or a minor allele frequency <5% in any tissue. A gene was considered expressed in a sample if its read count was greater than or equal to 6. Genes that were expressed in fewer than ten of the samples in a tissue were removed for the eQTL analysis in that tissue. To correct for varying degrees of admixture in the African-American subjects, we used the first three principal components of the genotyping data provided by the GTEx consortium and included these in our eQTL model.

We used the R MatrixEQTL package^20^ to calculate eQTLs with an additive linear model that included age, sex and ethnic background, as well as the first three genotype PCs as covariates:

$${\mathrm{Expression}} \sim {\mathrm{Genotype+Age+Sex+Ethnic}}\,{\mathrm{Background+PC}}{1}_{{\mathrm{genet}}}\\ + \, {\mathrm{PC}}{2}_{{\mathrm{genet}}}+{\mathrm{PC}}{3}_{{\mathrm{genet}}}+\epsilon$$

We tested for association between gene expression levels and SNPs both in *cis* and *trans*, where we defined *cis*-SNPs as those within 1 MB of the transcription start site of the gene based on mapping by using Bioconductor R biomaRt package.^21^ *P*-values were adjusted for multiple testing by using Benjamini–Hochberg correction for *cis*- and *trans*-eQTLs separately and only those with adjusted *P*-values < 0.2 were used in subsequent analyses.

### Community identification

For each tissue, we represented the significant eQTLs as edges of a bipartite network linking SNPs and gene nodes. For each network, we focused our analyses on the giant-connected component, which contained thousands of genes and tens of thousands of SNPs. Other connected components were excluded from the analyses due to their small size (each of them contained <50 genes and no more than 2 communities). To identify highly connected communities of SNPs and genes in the eQTL networks, we used the R condor package,^22^ which maximises the bipartite modularity.^23^ As recursive cluster identification and optimisation can be computationally slow, we calculated an initial community structure assignment on the weighted, gene-space projection, by using a fast unipartite modularity maximisation algorithm^24^ available in the R igraph package,^25^ then iteratively converged on a community structure corresponding to a maximum bipartite modularity.

The bipartite modularity is defined in Eq. (1), where $m$ is the number of links in the network, ${\widetilde{A}}_{ij}$ is the upper right block of the network adjacency matrix (a binary matrix where a 1 represents a connection between a SNP and a gene and 0 otherwise), ${k}_{i}$ is the degree of SNP $i$, ${d}_{j}$ is the degree of gene $j$ and ${C}_{i}$, ${C}_{j}$ the community indices of SNP $i$ and gene $j$, respectively.

1 $$Q=\frac{1}{m}\sum _{i,j}\left({\widetilde{A}}_{ij}-\frac{{k}_{i}{d}_{j}}{m}\right)\delta ({C}_{i},{C}_{j})$$

### Cancer-risk SNPs

We downloaded the NHGRI-EBI GWAS catalogue (accessed 24 April 2017, version v1.0) from the EBI website (https://www.ebi.ac.uk/gwas). We filtered associations with *P*-values > $5\times 1{0}^{-8}$ and extracted SNPs associated with a risk to develop cancer. We mapped the remaining SNPs to the GTEx data. Specifically, we determined LD blocks by using the plink2--blocks option with a 5 MB maximum block size^26^ and other options set to default values, which meant that two SNPs were considered in LD if the bottom of the 90% D-prime confidence interval was >0.70, and the top of the confidence interval was at least 0.98. We considered all SNPs in the same LD block as genome-wide significant cancer-risk SNPs.

### Cancer genes

We used information from two databases, the Network of Cancer Genes^27^ and the COSMIC census,^28^ to create a list of genes commonly mutated in cancers, or ‘cancer genes’ (Supplementary Table S2), including both oncogenes and tumour-suppressor genes. We mapped these cancer genes to the GTEx eQTL networks.

We tested whether cancer-risk SNPs are preferentially located in the promoters of the cancer genes. We downloaded transcription start site (TSS) positions for all genes present in the GTEx data from the Ensembl database^29,30^ and defined the promoters as the −750/+250-bp region around each TSS. We used Fisher’s exact test to determine whether the cancer gene promoters were enriched in cancer-risk SNPs. We used LD blocks rather than SNPs in this analysis to correct for linkage disequilibrium.

We also tested whether cancer-risk SNPs are more frequently associated with cancer genes than expected. In each network, we computed the ‘cancer degree' for each SNP by counting the number of significant cancer genes associated with each SNP based on our eQTL analysis. We compared the cancer degree distribution between cancer-risk and non-cancer-risk SNPs by taking into account the global degree distribution using $1{0}^{6}$ resamplings. We used the Mann–Whitney U test and compared *U*-values between the real and resampled data.

### Identifying eQTL community enriched for cancer-risk SNPs

We tested eQTL communities for enrichment of cancer-risk SNPs by using Fisher’s exact test. We defined cancer-risk LD blocks as those blocks containing cancer-risk SNPs. In each network, and for every cancer, we tested whether individual communities were enriched for risk SNPs, by using the whole network as background. To consider a community as enriched in cancer-risk SNPs, we used a threshold of a minimum of four LD blocks in the community.

### SNP core score calculation

We defined a SNP’s eQTL network core score as the SNP’s contribution to the modularity of its network community. For SNP $i$ in community $h$, its core score, ${Q}_{ih}$, is defined by Eq. (2). To normalise SNPs across communities, we accounted for community membership in our downstream testing (Eqs. (3) and (4)), which better accounts for community variation compared with the normalisation method used in ref. ^22^

2 $${Q}_{ih}=\frac{1}{m}\sum _{j}\left({\widetilde{A}}_{ij}-\frac{{k}_{i}{d}_{j}}{m}\right)\delta ({C}_{i},h)\delta ({C}_{j},h)$$

### Gene ontology functional category enrichment

We extracted the list of genes within each community in each tissue-specific network, and used the R GOstat package^31^ to perform a tissue-by-tissue analysis of the overrepresentation of Gene Ontology Biological Processes terms within each community enriched for cancer-risk SNPs. Our reference set consisted of all the genes present in the corresponding tissue-specific network. Communities were considered significantly enriched in a given category if the FDR-adjusted *P*-value was $\ < 0.05$.

### Cancer-risk SNP core score analysis

We compared the distribution of SNP core scores between cancer-associated SNPs fr and those not associated with traits or diseases for each tissue-specific network by using a likelihood ratio test (LRT). In our setting, the LRT assess whether a linear model that includes cancer-risk status (Eq. (4)) fits the observed data better than a linear model that does not include this variable (Eq. (3)). As the distribution of SNP core scores (${Q}_{ih}$) is not uniform across communities, we added community identity as a covariate in the linear regression. In Eqs. (3) and (4), ${Q}_{ih}$ is the core score of SNP $i$ in community $h$, $n$ the number of communities in the tissue. $I(GWAS=1)$ is an indicator function equal to 1 if the SNP is associated with a higher risk to develop cancer in GWAS and equal to 0 if it is not associated with any trait or diseases. SNPs associated with traits or diseases other than risk to develop cancer were filtered out. $I({C}_{k}=1)$ is an indicator function equal to 1 if the SNP belongs to community $k$ and equal to 0 otherwise.

3 $${Q}_{ih} \sim \sum _{k=1}^{n-1}I({C}_{k}=1)+\epsilon$$

4 $${Q}_{ih} \sim I(GWAS=1)+\sum _{k=1}^{n-1}I({C}_{k}=1)+\epsilon$$

To control for linkage disequilibrium between SNPs, we extracted the median of ${Q}_{ih}$ for cancer-risk SNPs and non-GWAS SNPs for each LD block, and used these values as input in the linear regressions.

## Results

### Cancer-risk SNPs are located in noncoding regions

We defined a set of cancer-risk SNPs based on the NHGRI-EBI GWAS catalogue (accession date: 2017-04-24); we extracted a set of 872 SNPs from 565 independent linkage disequilibrium (LD) blocks associated (at genome-wide significance $p\le 5\times 1{0}^{-8}$) with 135 unique traits and disease terms related to cancers, representing 41 cancer types (see Supplementary Table S3). Most of the cancer-risk SNPs were associated with only one cancer type; only 6% were associated with two or more cancers, and only 2% with more than three cancers. In contrast, most cancer types (82%) were associated with multiple independent SNPs, with the number of associated independent SNPs ranging between 1 (B-cell non-Hodgkin lymphoma, cardiac gastric cancer, chronic myeloid leukaemia, meningioma, non-melanoma skin cancer, small intestine neuroendocrine tumour and sporadic pituitary adenoma) and 95 (prostate cancer).

When examining the genomic location of cancer-risk SNPs, we found that their individual effect on the risk of developing cancer was also generally small with over 99% of cancer-risk SNPs having an odds ratio under 3. As observed for other traits and diseases,^32^ we found that only 9.7% of cancer-risk SNPs were exonic or splice variant SNPs, 40% were intronic and the rest was annotated as ‘regulatory variant’ or ‘intergenic.’ The lack of a clear known biological function based on SNP location suggests that many of the remaining 91.3% may play a regulatory role. To support this potential regulatory role, we found that 3.3% of cancer-risk SNPs are falling within a gene promoter defined as −750/+250 bp around a transcription start site (TSS), while only 0.9% of the non-cancer-risk SNPs are located in promoters (resampling *P*-value $p\le 1{0}^{-6}$). Moreover, cancer-risk SNPs are generally located nearby gene TSSs with 17.0% of them falling within 5 kb of a TSS and 84.0% within 100 kb, while only 8.0% and 25.4% of non-cancer-risk SNPs are located in these regions, respectively (resampling *P*-values $p\le 1{0}^{-6}$).

### Cancer-risk SNPs regulate cancer-related biological functions

To characterise the biological functions of this large number of small-effect, regulatory, cancer-risk SNPs, we performed a system-based eQTL analysis by using genotyping and RNA-Seq data from GTEx v6.0. After filtering and normalising the GTEx data, and eliminating tissues for which there were fewer than 200 samples, we were left with gene expression and genotype data for 13 tissues (12 primary tissues and 1 cell line, see Supplementary Table S1). We used MatrixeQTL,^20^ correcting for reported sex, age, ethnic background and the top three genotype principal components, to compute eQTLs in *cis* and *trans*, within a ±1- Mb window around the genes (see the ‘Methods' section). We used the same GTEx gene expression data and filtering steps as in our previous study,^33^ but added correction for four potentially confounding factors that have been shown to slightly impact the transcriptomic profile: the quality of extracted RNA, the place of death (at the accident site, during ambulance transfer, at hospital, etc.), the time interval between death and start of the tissue sampling and whether or not the donor was on a ventilator immediately prior to death. Despite these differences, the eQTL results largely correlated to those obtained previously (Spearman’s $\rho$ ranging from 0.99 to 1 when calculated by using β-values from the eQTL analysis, and from 0.89 to 0.94 when calculated by using *P*-values), and all conclusions from the previous papers were replicated.

For each of the thirteen tissues, we represented the significant *cis*- and *trans*-eQTLs as a bipartite network, where nodes are either SNPs or genes and edges are significant associations between SNPs and genes.^22,33^ To increase the size of the greatest connected component and because network centrality measures are more sensitive to false-negative than to false-positive edges,^34,35^ we relaxed the FDR cut-off and included all eQTLs with FDR q-values under 0.2. We obtained thirteen tissue-specific networks containing between 57,641 (ATA—aorta) and 431,036 (THY—thyroid) SNPs (median across all 13 tissues = 198,226), corresponding to between 3550 and 34,016 LD blocks (median = 15,514) and between 1090 and 10,003 genes (median = 4820).

We used the R condor package^22^ in each of the thirteen eQTL networks to identify communities, defined as groups of SNPs and genes more densely connected to each other than would be expected by chance (see the Methods section). The bipartite modularity measures whether the network is structured in communities in which genes and SNPs are more likely to be linked to other members of their community than to the rest of the network, ranging from 0.83 to 0.97 (median = 0.95). It indicates that these networks are highly modular, with SNPs and genes grouped in well-defined communities. In each of the thirteen tissues, we found between 29 and 177 (median = 124) communities. We then functionally annotated those communities by testing for overrepresentation of genes annotated to Gene Ontology (GO) biological processes^36^ (Supplementary Table S4). We found that some communities were enriched for genes involved in biological functions shared across the thirteen tissues (immunity, gene expression regulation and rna metabolism), while others were tissue-specific (such as heart muscle contraction in heart left ventricle and smooth muscle contraction in oesophagus muscularis, which is a smooth muscle). Gene Ontology enrichment and network modularity are similar to those found in Fagny et al.^33^

**Fig. 1:**
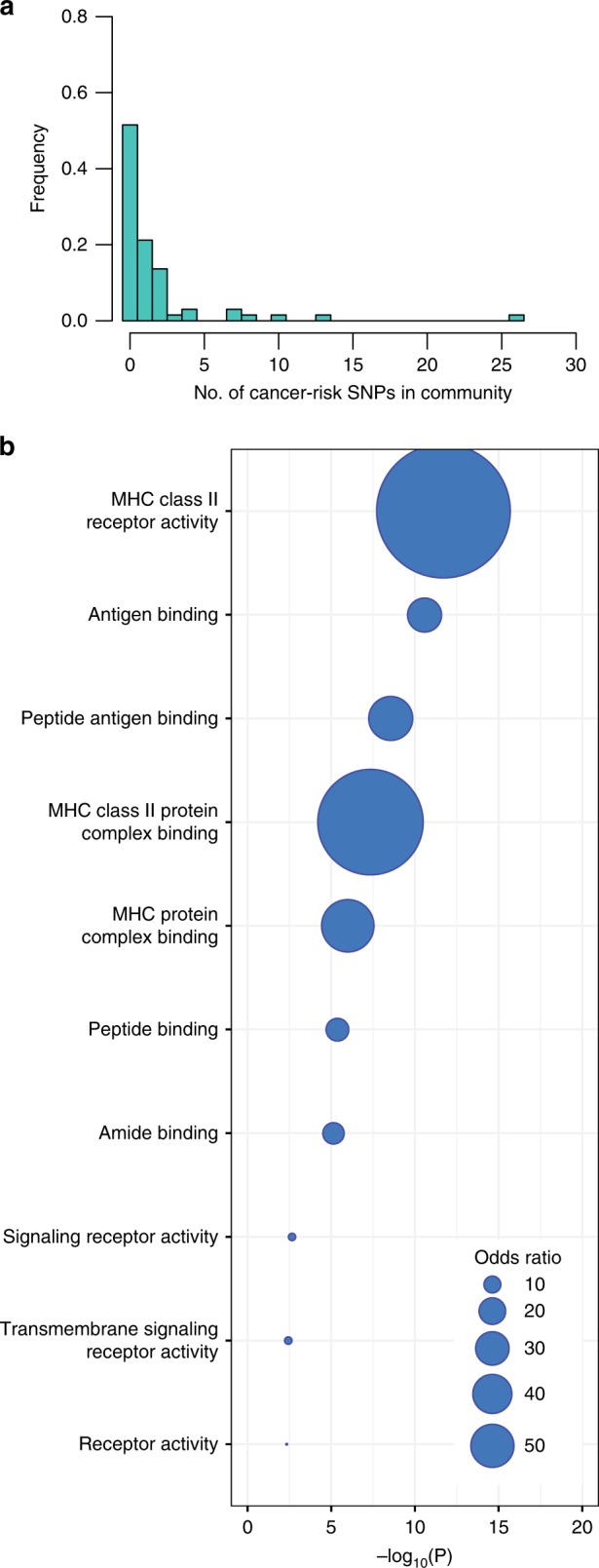
Cancer-risk SNPs are distributed across the network communities and functional roles. **a** Distribution of cancer-risk SNPs in each community in whole blood. **b** Gene Ontology Term enrichment for communities in community 13 of the whole-blood eQTL networks that is also enriched for cancer-risk SNPs

We mapped the cancer-risk SNPs to the eQTL network for each of the 13 tissues. Of the 872 cancer-risk SNPs, 582 were either an eQTL or in strong linkage disequilibrium (${r}^{2}\ > \ 0.8$, see Methods) with an eQTL to at least one gene in at least 1 of the 13 tissues, confirming the regulatory role of these SNPs. In 9 out of 13 tissues, these cancer-risk eQTLs were slightly enriched for *trans*-eQTLs compared with the non-cancer-risk eQTLs, with odds ratios ranging from 0.91 in the heart left ventricle (Fisher test $p=1.00$) to 7.84 in thyroid (*P* = 9.51 × 10^−49^, Supplementary Table S5). Among these 582 cancer-risk SNPs, 512 mapped to the network giant-connected component (either directly or through membership in a strong LD block) in at least one tissue. These SNPs map to communities that are associated with a wide range of biological processes. Depending on the tissue, between 21% (heart left ventricle) and 49% (lung) of communities contain at least one cancer-risk SNP. However, most communities contain only one or two cancer-risk SNPs (Table 1 and Fig. 1a). A complete list of the cancer-risk SNPs mapping to the communities in each of the 13 tissues and their corresponding Gene Ontology Biological processes is provided in Supplementary Table S6. A searchable version of these results is provided at http://networkmedicine.org:3838/cancer_eqtl/.

**Table 1:** Communities enriched in cancer-risk SNPs

Tissue	Abbrev	# Communities	# With cancer risk SNPs	# Enriched in cancer risk SNPs^a^	# Enriched in 1+ cancer type^b^
Adipose subcutaneous	ADS	82	39	4	1
Aorta	ATA	29	13	2	2
Artery tibial	ATT	95	40	2	0
Fibroblast	FIB	156	54	2	3
Oesophagus mucosa	EMC	147	45	4	2
Oesophagus muscularis	EMS	143	58	2	1
Heart left ventricle	HRV	124	26	2	2
Lung	LNG	35	17	4	1
Skeletal muscle	SMU	86	33	3	1
Tibial nerve	TNV	152	64	8	1
Skin	SKN	163	71	4	3
Thyroid	THY	177	77	6	2
Whole blood	WBL	66	32	5	2

^a^In this column, all cancer risk SNPs across all cancer types were pooled together under a “cancer risk” label before the enrichement analysis was performed.

^b^In this column, each cancer type was analysed separately in each community. One community can be enriched for risk SNPs for multiple cancers

We then tested each community in each tissue for enrichment of cancer-risk SNPs. Because studies have shown that GWAS top hits are not always causal SNPs, and that they often do not correspond to the strongest eQTL hit, we included all SNPs in LD with a cancer-risk SNP in our enrichment analyses. We pooled SNPs from the same LD block and annotated them as cancer-risk LD blocks or not cancer-risk LD blocks. We used these LD blocks for all enrichment tests. By using Fisher’s exact test, we identified 2–8 (median = 4) communities in each tissue that were enriched for cancer-risk SNPs (all cancers pooled together), and only a very small number that were enriched for cancer-risk SNPs associated with one particular type of cancer (Table 1). The details about enrichment, odds ratios and *P*-values for each cancer type, each community and each tissue are given in Supplementary Table S7.

We explored the functional consequences of cancer-risk SNPs by testing whether communities enriched for these SNPs were also enriched for genes annotated to GO biological process terms. Across all tissues except tibial artery (ATA), we found that communities with increased representation of cancer-risk SNPs contain genes enriched in functions linked to immunity, mainly genes belonging to the major histocompatibility complex (MHC) class I and II families, and that the majority of these immune-related genes were *cis*-eQTLs with cancer-risk SNPs. An example of the Gene Ontology enrichment of this shared community in whole blood is presented in Fig. 1b. Other communities were enriched in nonspecific biological processes like RNA metabolic processes and DNA binding. Only two of the tissue-specific networks presented a community enriched in both cancer-risk SNPs and tissue-specific biological pathways: epithelium development in skin and cell–cell adhesion in fibroblasts (Supplementary Table S4).

### Cancer-risk SNPs are core SNPs in their communities

**Fig. 2:**
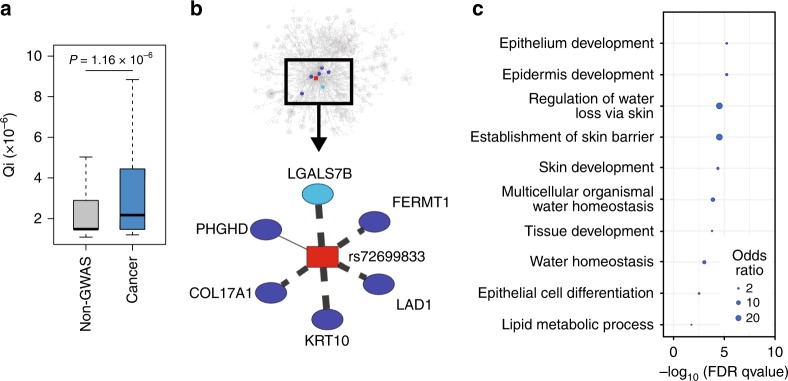
Network properties of GWAS cancer-risk SNPs. **a** Distribution of core scores for SNPs associated with increased cancer risk in skin by GWAS (in blue) and other skin SNPs (in grey). *P*-values were obtained by using a likelihood ratio test and pruning for SNPs in linkage disequilibrium. Distributions for all tissue-specific networks are shown in Supplementary Fig. S1. **b** An example of a SNP with high core score: rs72699833, in LD with rs11249433, a SNP associated with a higher risk to develop breast cancer. This SNP belongs to community 147 (top panel), which is enriched for breast cancer-risk SNPs and is associated with multiple genes involved in epithelium development. LGALS7B is represented here but belongs to another community (107). Details on the associations are provided in Supplementary Table S8. Dashed lines indicate association in *trans*, full line in *cis*. The thickness of the lines corresponds to the strength of the association. **c** Enrichment in Gene Ontology Terms for community 147 in the skin

As shown previously, the communities in eQTL networks have a characteristic structure, with local hubs, or ‘core SNPs,’ central within their communities. Disease-associated SNPs found through GWAS have been shown to map not only to communities with relevant biological functions, but also to the cores of those communities.^22,33^ As a measure of SNP centrality, we define a ‘core score’ equal to the relative modularity contributed by a SNP to the overall modularity of its community (see Eq. (2) in the Methods section). We calculated core scores for all SNPs in the network and compared the core score distribution of cancer-risk SNPs and SNPs not associated with any trait or disease in GWAS. We found that cancer-risk SNPs were enriched for higher core scores (Fig. 2a for skin and Supplementary Fig. S1 for other tissues). This result is consistent across tissues, indicating that germline cancer-risk SNPs, being central to their communities, affect the expression of many genes involved in coherent biological processes related to cancer development and progression.

For example, SNP rs72699833 is a core SNP in skin community 147. This SNP is in LD with rs11249433, which has been associated with an increased risk of breast cancer (Fig. 2b). By examining skin community 147, we find enrichment for SNPs associated with breast cancer (Supplementary Table S7) and for genes involved in epithelium development (Supplementary Table S4 and Fig. 2c); as breast cancer is an epithelial cancer, the association with skin is not surprising. SNP rs72699833 is located on chromosome 1 and is associated in *cis* with *PHGDH*, a gene involved in the metabolism of serine that is overexpressed in some subtypes of breast, cervical, colorectal and non-small-cell lung cancer, and in these diseases generally associated with a poorer outcome.^37–40^

In addition, rs72699833 is associated through our eQTL analysis in *trans* with five other genes: *LAD1* on chromosome 1, *COL17A1* on chromosome 10, *KRT10* on chromosome 17, *LGALS7B* on chromosome 19 and *FERMT1* on chromosome 20 (Supplementary Table S8). All of these genes are involved in epithelium development and in particular with extracellular matrix (ECM) secretion and cell–ECM interactions. Most of these genes have been shown to be dysregulated in breast cancer or during epithelial–mesenchymal transition. Indeed, *LAD1* has been associated with aggressive breast tumours,^41^ *COL17A1* is underexpressed in breast cancer and overexpressed in head and neck squamous cell carcinoma, lung squamous cell carcinoma and lung adenocarcinoma^42^ and *FERMT1* is a known mediator of epithelial–mesenchymal transition in colon cancer.^43^

### Cancer-risk SNPs preferentially target cancer genes

We expected that cancer-risk SNPs might be preferentially associated with genes known to be involved in cancer development and progression. We assembled a catalogue of oncogenes and tumour-suppressor genes ('cancer genes') by using databases that included the Network Gene Cancer version 5.0^27^ and the COSMIC^44^ census (see Methods and Supplementary Table S2).

**Fig. 3:**
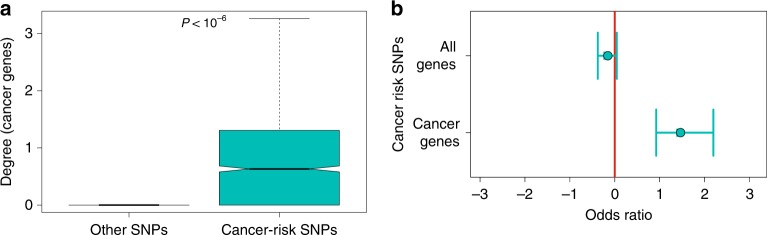
Cancer-risk SNPs are preferentially located in the promoters of cancer genes. **a** Cancer-risk SNPs preferentially target oncogenes and tumour-suppressor genes across all tissues. Box plots present distributions of a number of tumour-suppressor genes and oncogenes targeted by cancer-risk SNPs and other SNPs. The *P*-value was obtained by using $1{0}^{6}$ resamplings, by taking into account global differences in degree distribution between cancer-risk SNPs and other SNPs. This indicates that cancer genes are likely associated with one or more cancer-risk SNPs, but not other eQTL SNPs. The same analysis for each tissue-specific network is presented in Supplementary Fig. S2. **b** Cancer-risk SNPs are preferentially located in the promoters of oncogenes and tumour-suppressor genes relative to other genes. This figure shows the odds ratio for finding cancer-risk SNPs, rather than other SNPs, in promoters of all genes’ promoters (top) or oncogenes and tumour-suppressor genes’ promoters (bottom). The same analysis for each tissue-specific eQTL network is presented in Supplementary Fig. S3

We tested whether cancer-risk SNPs are more frequently associated with cancer genes than other SNPs based on the eQTL networks. We mapped cancer-risk SNPs to the giant-connected component of each of the thirteen tissue-specific eQTL networks. We then compared the number of cancer genes targeted by cancer-risk SNPs and other SNPs, by taking into account linkage disequilibrium and global degree distribution (the total number of genes to which they were associated; see Methods). We showed that cancer-risk SNPs were indeed more likely to target cancer genes than expected by chance ($p\ <\ 1{0}^{-6}$ based on 1,000,000 resamplings) when studying all networks together (Fig. 3a); similar results were found in each tissue-specific network (Supplementary Fig. S2).

Finally, we tested whether the cancer-risk SNPs are located in the promoters of genes known to be mutated in cancers. For genes expressed in at least 1 of the 13 tissues, we mapped SNPs with minor allele frequencies >5% to their promoters. We then compared SNPs mapping to cancer and non-cancer genes. We found that cancer-risk SNPs are not more likely than other SNPs to fall in promoter regions of non-cancer genes, but cancer-risk SNPs appear preferentially in the promoters of oncogenes and tumour-suppressor genes (Fig. 3b, Supplementary Fig. S3).

## Discussion

It has long been known that both germline and somatic mutations in oncogenes and tumour-suppressor genes drive development and progression of cancer.^45^ However, we know that cancer has a genetic component beyond these well-known ‘cancer drivers,’ and that genetic factors can influence differences in the natural history of cancer in individuals possessing the same somatic mutations. Genome-wide association studies have analysed hundreds of thousands of individuals to find genetic variants that are associated with increased risk of developing cancer, but many of these fall into intergenic regions and have no clear functional association with cancer drivers. As a result, the functional link between genetic risk and the mechanism of cancer development has not been fully understood.

By using the data from GTEx, we built bipartite eQTL networks representing germline SNP–gene associations, including both *cis-* and *trans-*acting eQTLs in 13 different tissues by using an extension of a method we had used previously.^33^ When we mapped germline cancer-risk SNPs to each of these networks, we found that cancer-risk SNPs are associated with the expression levels of oncogenes and tumour-suppressor genes at a far greater rate than expected by chance. This indicates not only that mutations in these cancer genes are important, but also that the genetic control of these genes by regulatory variants plays an important role. A natural assumption might be that cancer-risk SNPs lie in the promoter regions of oncogenes and tumour-suppressor genes, but many of the GWAS cancer-risk SNPs are located outside of promoters, leaving the question of the mechanism by which these variants exert their influence.

As we reported previously, SNP–gene eQTL networks are organised into highly modular, regulatory communities that are frequently enriched for genes carrying out distinct biological functions. Consistent with our previous analysis of disease-associated SNPs,^22,33^ we find that cancer-risk SNPs are overrepresented at the ‘cores' of individual communities, meaning that those SNPs are at key positions in functional communities where the cancer-risk SNPs can influence the expression of groups of functionally related genes, thus exerting a substantial effect on key biological processes.

Despite the observed concentration of GWAS SNPs in the core of communities, we find that disease-associated germline SNPs in cancer and chronic diseases are distributed differently across eQTL network communities. In chronic obstructive pulmonary disease (COPD), GWAS SNPs map to a small number of communities that possess disease-relevant functions.^22^ In contrast, we find that cancer-risk SNPs are distributed across a large number of functionally diverse communities; this distribution is consistent with our understanding that cancer is a systemic disease that affects many different cellular processes.

When we search for communities with the greatest enrichment of cancer-risk SNPs across all thirteen GTEx tissues, we find an overrepresentation of these SNPs in communities enriched for immune-related genes. In particular, cancer-risk SNPs are linked to altered expression of MHC class I and II genes. MHC genes are clustered on the p-arm of chromosome 6, and play a role in recognising pathogen-infected and other types of modified cells (including cancer cells) and in triggering the innate and adaptive immune system. It is well known that the power of eQTL studies to detect associations between genotype and gene expression depends on the minor allele frequency.^46,47^ In this study, we used the data from 13 tissues for which we had available matching RNA-seq and genotyping data in 200 or more samples; the sample sizes vary between 212 (HRV—heart left ventricle) and 378 samples (SKN—skin). Even the largest sample size does not allow us to reach the maximum power of eQTL detection for alleles with low–intermediate frequencies (0.1–0.2), and so our results are likely to be enriched for high–intermediate-frequency alleles (0.2–0.5). Because the MHC region is known to include many SNPs with high minor allele frequencies,^48^ we may be overestimating the role of genes associated with cancer risk relative to other loci. Further, the high recombination rate and high density of SNPs and genes in the MHC region makes association studies difficult. However, most of the eQTL associations in the region are in *cis*, and some of these have been found in previous studies that targeted the MHC region,^49–51^ lending support to our findings. By modulating the expression of MHC genes, cancer-risk SNPs may be modifying an individual’s immune response so as to interfere with the elimination of mutated, pre-cancer cells. Indeed, those eQTL-associated immune response genes belong to the MHC class I and II regions that are known to be downregulated in most cancer cells and affect genes that are targets for some of the newest cancer therapies.^52,53^

In addition to the association with immune response observed in all thirteen tissues, cancer-risk SNPs are overrepresented in other functionally interesting communities. For example, SNPs have been linked in GWASes to breast and epithelial cancer cluster in one eQTL network community in the skin network, a community that is enriched for genes linked to epithelium development and extracellular matrix secretion. These and other examples suggest that the distribution of these SNPs within and among communities provides evidence for the functional significance of germline SNPs that are associated with cancer risk and development. It is particularly notable that while the cancer-risk SNPs that associate with gene expression differ between tissues, those diverse SNPs are generally associated through the eQTL network community structure with common functions across tissues. This suggests that similar mechanisms, moderated by tissue-specific expression, may be perturbed across many cancers. This, in turn, may well point to common disease-associated functions that could be targeted therapeutically.

Representing eQTLs by using a bipartite network in thirteen tissues, we find that SNPs and genes are organised into communities that reflect the genetic regulatory influence of SNPs on functionally related groups of genes, as demonstrated by GWAS annotation, gene ontology analyses and enrichment of cancer-risk SNPs in the promoters of cancer genes. By mapping disease-risk SNPs to these networks, we can develop hypotheses about how these SNPs work both individually and collectively to moderate risk and possibly enable disease development.

Our analysis identified significant regulatory roles for noncoding SNPs acting in both *cis* and *trans*. Non-genic SNPs have long been known to affect gene expression by altering transcription factor-binding sites. We also know that non-genic variants outside of promoter regions can influence gene expression by modifying long-range chromatin interactions between distal *cis*-regulatory elements known as enhancers and their target genes through modification of 3D chromatin folding.^54^ Our analysis suggests that the regulatory effects of cancer-risk SNPs influence both cancer genes and other genes that control processes associated with diverse processes, including development and immune response. Indeed, long-range regulatory effects altering enhancers have been shown to play a role in obesity and Parkinson’s disease,^55,56^ and several examples of altered interactions between enhancers and their target genes, leading to oncogenesis, have been described.^57^

This study provides the first systematic analysis of eQTLs by using network methods to explore the regulatory role of germline cancer-risk SNPs. By mapping cancer-risk SNPs to bipartite networks built from both *cis*- and *trans*-eQTLs in thirteen tissues, we show that cancer-risk SNPs play a distinctive role in defining the structure of such networks. Cancer-risk SNPs are associated not only with cancer genes, but with many other genes associated with biological functions that can be linked to cancer development and progression. The clustering of cancer-risk SNPs and relevant genes into highly modular communities provides a framework into how these SNPs moderate the risk of cancer development. While we tend to think of cancer development in terms of drivers, our analysis indicates that the effects of these drivers are likely moderated through the interactions of those genes with regulatory variants that can increase, or decrease, cancer risk.

It is worth noting that the approach we present here can also be used to explore the functional roles played by other SNPs, linked to disease or other processes through GWASes. While the analysis of eQTL networks does not fully bridge the gap between genotype and phenotype, it provides an explanatory framework that can be used to further investigate the genetic risk of disease and the synergistic effects of germline genetic variants.

## Supplementary information

Supplementary Information

Supplementary Tables S2, S3, S4, S6, S7

## Data Availability

All genotyping and gene expression data were downloaded from the dbgap repository (accession number phs000424.v6.p1). GWAS results were downloaded from the EBI/NGRHI GWAS catalog website (v1.0, accessed 8 December 2015). eQTL networks and a browsable version of our results were made available on the following website: http://networkmedicine.org:3838/cancer_eqtl/.
