# Supplementary material for: Nongenic cancer-risk SNPs affect oncogenes, tumour-suppressor genes, and immune function
Source: Br J Cancer. 2019 Dec 6;122(4):569–77. doi: 10.1038/s41416-019-0614-3 (PMC7028992; doi:10.1038/s41416-019-0614-3)
Supplement: Supplementary file 1 — Supplementary Information [file 41416_2019_614_MOESM1_ESM.pdf]

Supplementary Information

Nongenic cancer-risk SNPs affect oncogenes, tumor suppressor genes and immune function

M. Fagny[1], J. Platig[2,3], M.L. Kuijjer[4,5,6], X. Lin[5], and J. Quackenbush[2,4,5,7]

[1]Genetique Quantitative et Evolution - Le Moulon, Institut National de la Recherche agronomique, Universite Paris-Sud, Centre National de la Recherche Scientique, AgroParisTech, Universite Paris-Saclay, France

[2]Channing Division of Network Medicine, Brigham and Women's Hospital, Boston, Massachusetts, USA

[3]Harvard Medical School, Boston, Massachusetts, USA

[4]Department of Biostatistics and Computational Biology, Dana-Farber Cancer Institute, Boston, Massachusetts, USA

[5]Department of Biostatistics, Harvard T.H. Chan School of Public Health, Boston, Massachusetts,USA

[6]Centre for Molecular Medicine Norway, University of Oslo, Oslo, Norway

[7]Department of Cancer Biology, Dana-Farber Cancer Institute, Boston, Massachusetts, USA

Supplementary Figures

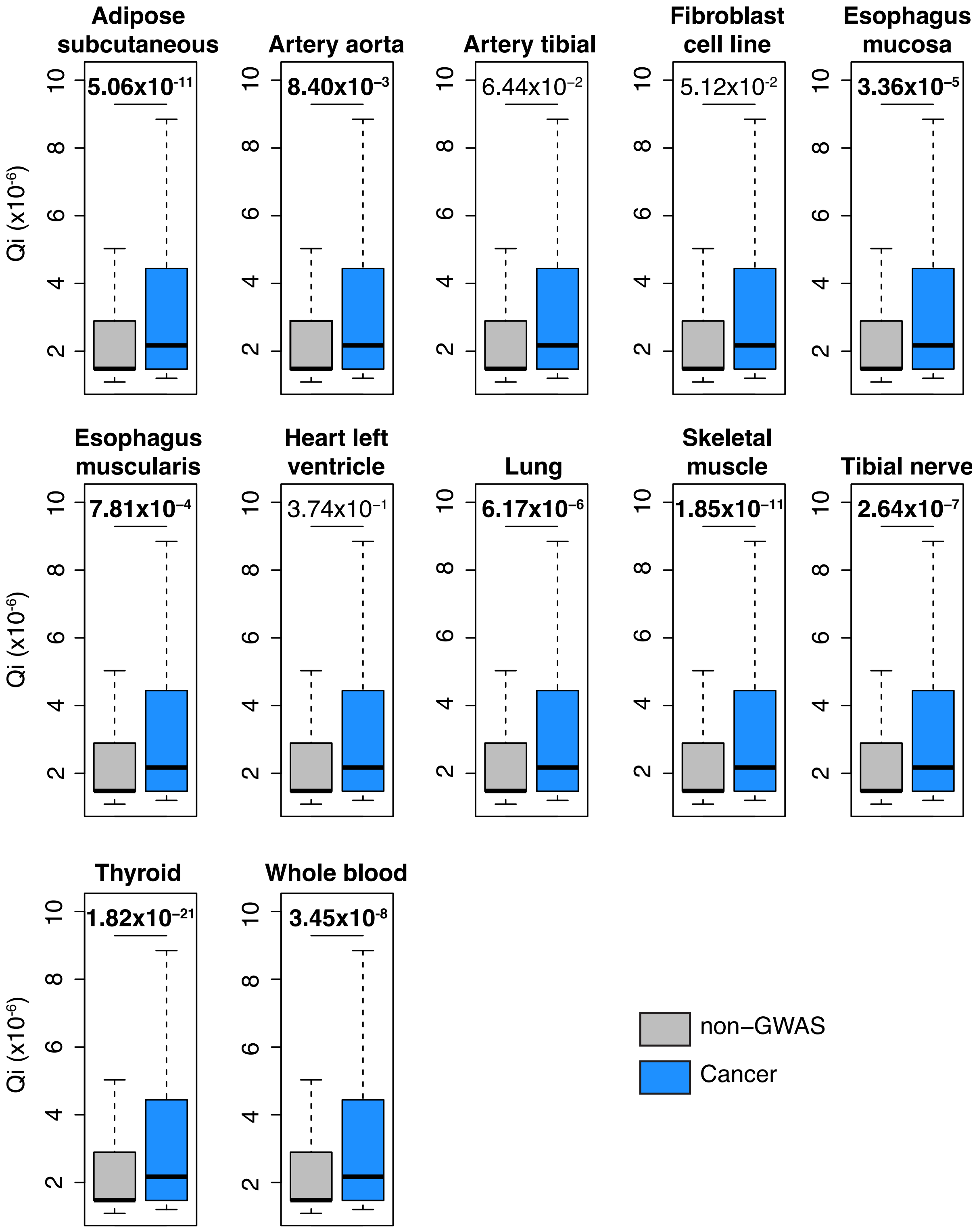

Figure S1: Related to Figure 2. SNPs associated in GWAS with a increased risk of developing cancer have higher network core scores than non-cancer SNPs. This enrichment is significant in ten of thirteen tissues; significant p-values are indicated in bold text. The lack of significance in tibial artery and heart left ventricle may reflect the rarity of cancers in these tissues. The failure to reach significance in fibroblast cell lines may be due to alteration of normal cellular functions in culture.

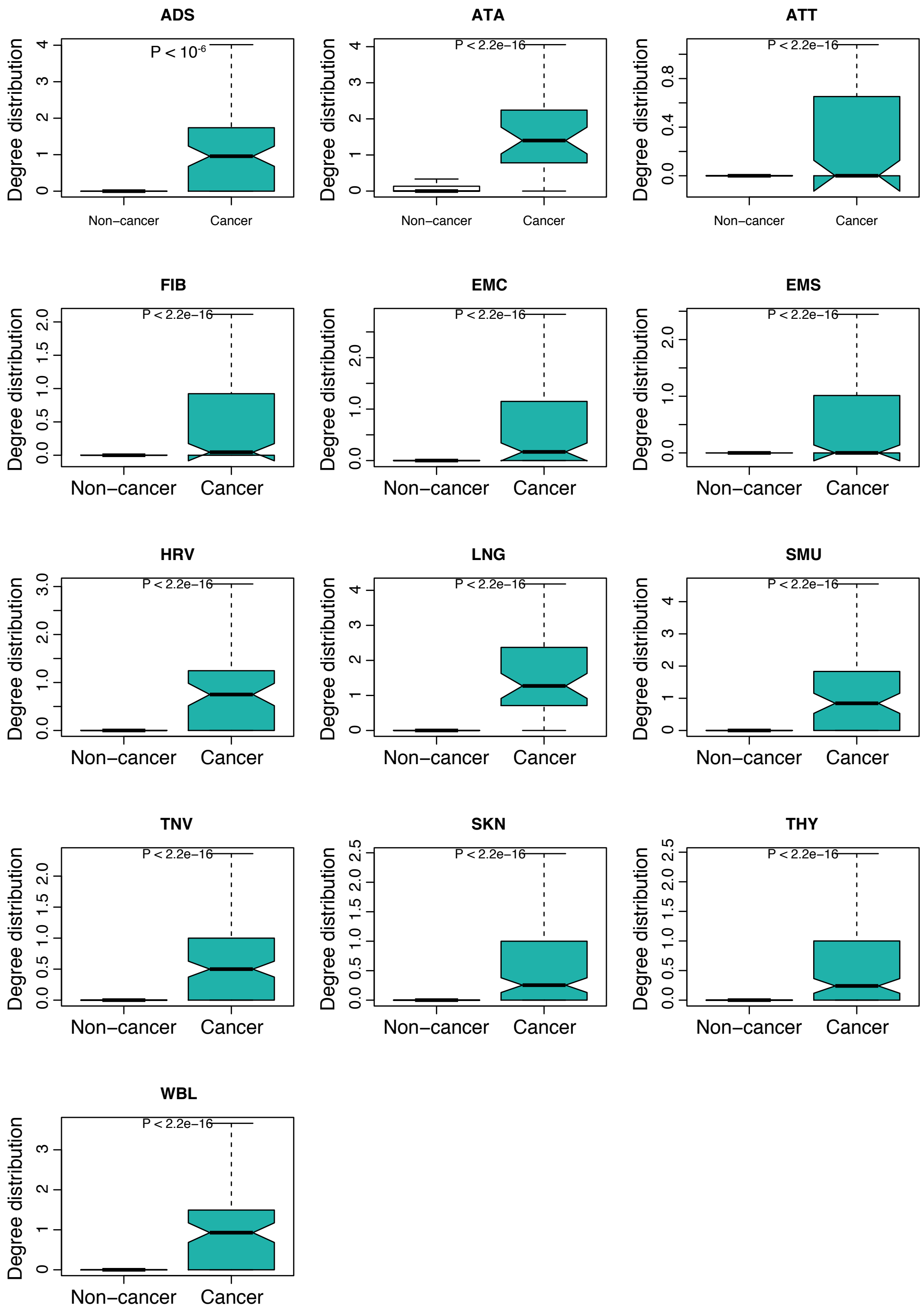

Figure S2: Cancer risk SNPs preferentially target oncogenes and tumor suppressor genes. In each panel, box plots present distributions of the number of tumor suppressor genes and oncogenes associated with cancer-risk SNPs and with other SNPs in each tissue-specific network. P values were obtained using 10^6 resamplings, taking into account global differences in degree distribution between cancer-risk SNPs and other SNPs.

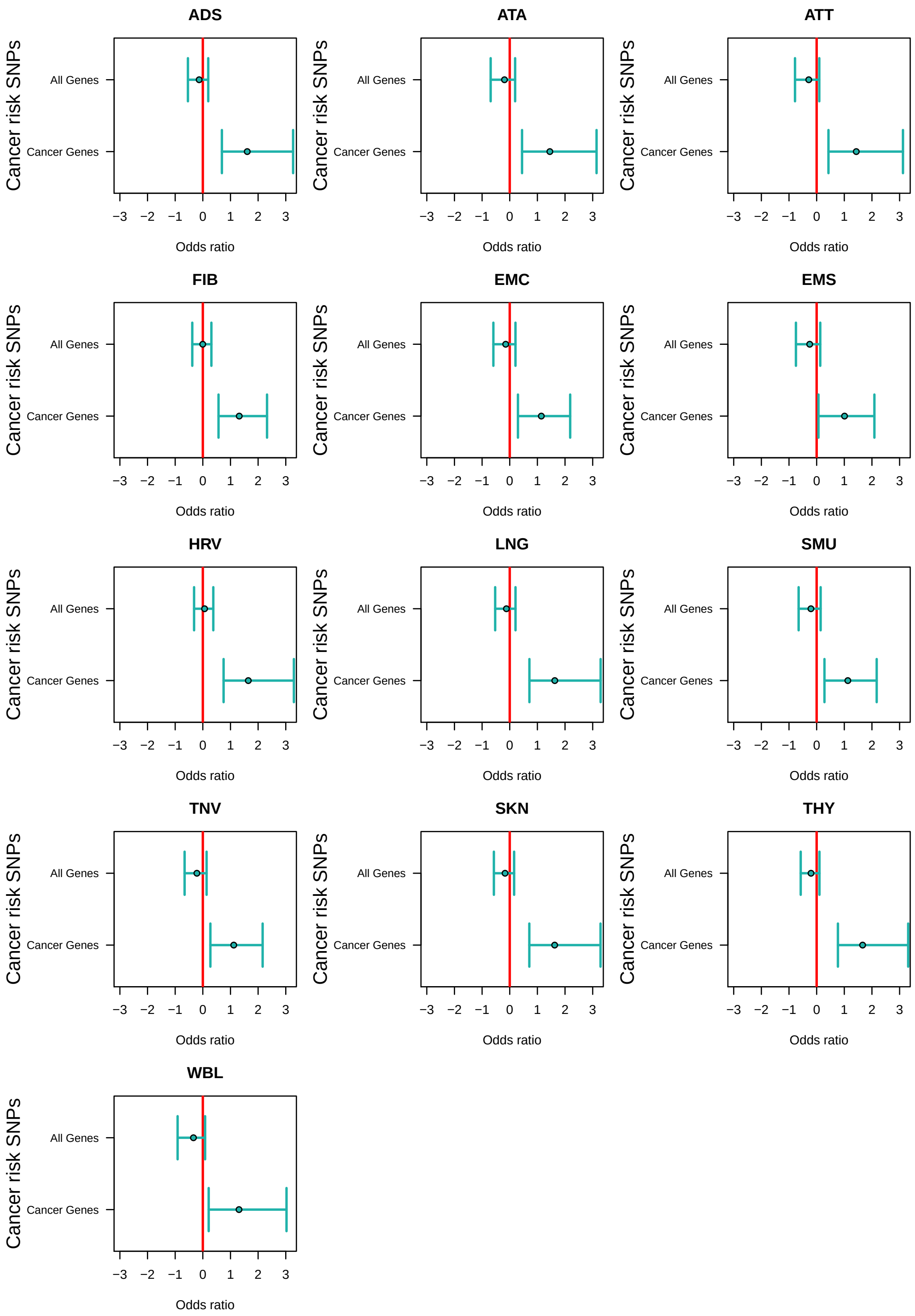

Figure S3: Cancer-risk SNPs associated with gene expression levels in eQTL analyses are preferentially located in the promoters of oncogenes and tumor suppressor genes, independent of tissue. The Odds ratio measures the enrichment in cancer-risk SNPs among all genes' promoters (top) or oncogenes and tumor suppressor genes' promoters (bottom) compared to other SNPs.

Supplementary Tables

Table S1: Summary of data: tissues and number of samples.

Tissue names	Abbreviation	GTEx tissues	Samples
Adipose subcutaneous	ADS	Adipose - Subcutaneous	313
Aorta	ATA	Artery - Aorta	216
Artery tibial	ATT	Artery - Tibial	303
Fibroblast	FIB	Skin cells - Transformed fibroblasts	291
Esophagus mucosa	EMC	Esophagus - Mucosa	276
Esophagus muscularis	EMS	Esophagus - Muscularis	245
Heart left ventricle	HRV	Heart - Left ventricle	212
Lung	LNG	Lung	290
Skeletal muscle	SMU	Muscle - Skeletal	378
Tibial nerve	TNV	Nerve - Tibial	278
		Skin - Not sun exposed (Suprapubic)	127
Skin	SKN	Skin - Sun exposed (Lower leg)	243
		Total	370
Thyroid	THY	Thyroid	295
Whole blood	WBL	Whole blood	365

Table S2: Tumor suppressor genes and Oncogenes IDs.

Table S3: List of cancer-related terms in EBI-GWAS catalog.

Table S4: Gene Ontology enrichment for communities.

Table S5: Enrichment in trans-eQTLs among cancer-risk SNPs.

Tissue	Odds ratio	P value
**ADS**	1.77	4.64E-02
**ATA**	2.09	4.56E-02
**ATT**	1.88	4.29E-02
**FIB**	2.40	1.37E-03
**EMC**	1.59	7.81E-02
**EMS**	1.46	2.14E-01
**HRV**	0.91	1.00E+00
**LNG**	2.11	1.35E-02
**SMU**	2.33	8.38E-04
**TNV**	1.94	1.07E-02
**SKN**	1.40	1.79E-01
**THY**	7.84	9.51E-41
**WBL**	2.19	6.16E-03

Table S6: Cancer-risk SNPs mapping to communities and corresponding Gene Ontology enrichment.

Table S7: Enrichment in cancer-risk SNPs among communities.

Table S8: An eQTL example : genes associated with rs72699833

Ensembl ID	HGNC	Chr	Start	End	Type	t.stat	P	FDR
ENSG00000092621.7	**PHGDH**	1	120,202,421	120,286,838	cis	-3.80	1.67E-04	2.97E-02
ENSG00000159166.9	LAD1	1	201,342,372	201,368,736	trans	-5.62	3.73E-08	1.93E-01
ENSG00000065618.12	COL17A1	10	105,791,044	105,845,760	trans	-5.61	3.97E-08	1.99E-01
ENSG00000186395.6	**KRT10**	17	38,974,369	38,978,847	trans	-6.02	4.24E-09	4.12E-02
ENSG00000178934.4	**LGALS7B**	19	39,279,851	39,282,389	trans	-6.00	4.84E-09	4.60E-02
ENSG00000101311.11	FERMT1	20	6,055,492	6,104,191	trans	-5.70	2.54E-08	1.53E-01
